# *Escherichia coli* DNA ligase B may mitigate damage from oxidative stress

**DOI:** 10.1371/journal.pone.0180800

**Published:** 2017-07-11

**Authors:** Truston J. Bodine, Michael A. Evangelista, Huan Ting Chang, Christopher A. Ayoub, Buck S. Samuel, Richard Sucgang, Lynn Zechiedrich

**Affiliations:** 1 Interdepartmental Program in Translational Biology and Molecular Medicine, Baylor College of Medicine, Houston, TX, United States of America; 2 Department of Molecular Virology and Microbiology, Baylor College of Medicine, Houston, TX, United States of America; 3 Medical Scientist Training Program, Baylor College of Medicine, Houston, TX, United States of America; 4 Verna and Marrs McLean Department of Biochemistry and Molecular Biology, Baylor College of Medicine, Houston, TX, United States of America; 5 Department of BioSciences, Rice University, Houston, TX, United States of America; 6 Alkek Center for Metagenomics and Microbiome Research, Baylor College of Medicine, Houston, TX, United States of America; 7 Department of Pharmacology, Baylor College of Medicine, Houston, TX, United States of America; University of South Alabama Mitchell Cancer Institute, UNITED STATES

## Abstract

*Escherichia coli* encodes two DNA ligases, ligase A, which is essential under normal laboratory growth conditions, and ligase B, which is not. Here we report potential functions of ligase B. We found that across the entire Enterobacteriaceae family, ligase B is highly conserved in both amino acid identity and synteny with genes associated with oxidative stress. Deletion of *ligB* sensitized *E*. *coli* to specific DNA damaging agents and antibiotics resulted in a weak mutator phenotype, and decreased biofilm formation. Overexpression of *ligB* caused a dramatic extension of lag phase that eventually resumed normal growth. The ligase function of ligase B was not required to mediate the extended lag phase, as overexpression of a ligase-deficient *ligB* mutant also blocked growth. Overexpression of *ligB* during logarithmic growth caused an immediate block of cell growth and DNA replication, and death of about half of cells. These data support a potential role for ligase B in the base excision repair pathway or the mismatch repair pathway.

## Introduction

Bacteria have evolved multiple pathways to repair DNA damage in response to the pressure of constant genomic insult [1]. DNA repair invariably ends with ligation of the DNA phosphate backbone, making ligases of utmost importance. Most bacteria encode one NAD^+^-dependent DNA ligase and one to several ATP-dependent DNA ligases [2,3]. Ligase A is highly conserved in bacterial genomes; it retains similar domain structure, length (656–837 amino acids), and a three-step nucleotidyl transfer reaction [2]. As an essential and well conserved enzyme, Ligase A is an attractive target for antibiotic development [4].

*E*. *coli* was the first organism found to encode two NAD^+^-dependent DNA ligases and it encodes no known ATP-dependent ligases [2]. Purified ligase B can ligate the phosphate backbone of DNA, but with less than 1% the *in vitro* activity of purified ligase A [2]. Ligase B is predicted to have similar protein structure to ligase A but lacks the BRCA-like C-terminal domain and also lacks two of the four tetracysteine zinc-finger motifs [2]. These missing domains, however, do not account for the reduced *in vitro* activity because ligase B-ligase A hybrids that contain these domains do not have increased ligase activity [2].

It is reported in the literature that in *E*. *coli* expression of the *ligB* gene increases by at least two-fold in response to cold shock [5], oxidative stress [5], cadmium at pH 7 [6], formation of a biofilm, or in older biofilms [7,8]. In contrast, evolutionary adaptation to high temperature [9] or heat shock [10] decreases *ligB* expression.

An allelic variation of *ligB* is tightly associated with fluoroquinolone resistance in *E*. *coli* clinical isolates [11]. Every fluoroquinolone-resistant *E*. *coli* clinical isolate evaluated thus far encodes the same non-synonymous single nucleotide polymorphism, which encodes an amino acid cap found at the N-terminal α-helix of a helix-hairpin-helix domain involved in DNA binding [11]. An *E*. *coli ligB* null strain complemented with the fluoroquinolone resistance-associated *ligB* allele responds differently to hydrogen peroxide or ultraviolet (UV) irradiation from the *ligB* null complemented with the *ligB* allele not associated with fluoroquinolone resistance [11]. These data, in conjunction with alteration of *ligB* expression in response to cell stressors, indicate a possible role for ligase B in the bacterial stress response, possibly by helping the cell respond to and/or deal with DNA damage.

## Materials and methods

### Chemicals and reagents

[Methyl-^3^H]-thymidine was from Amersham Pharmacia. Isopropyl β-D-1-thiogalactopyranoside (IPTG), cadmium sulfate, bleocin, mitomycin C, ciprofloxacin, crystal violet, and rifampicin were from Sigma-Aldrich. Bovine liver catalase was from Worthington Biochemical. E-Test strips were obtained from Biomérieux. LIVE/DEAD BacLight Bacterial Viability Kit was from Molecular Probes. All other chemicals were purchased from VWR.

### Analysis of ligase B sequences

DNA and protein sequences were obtained from the Pathosystems Resource Integration Center (PATRIC) [12] and were managed in Geneious (version 5.6.6 published by BioMatters, Ltd.). Global amino acid alignments were performed using ClustalW [13], and the phylogenetic tree was built with the Jukes-Cantor genetic distance model [14] and neighbor-joining tree building method [15].

### Strain construction

The Δ*ligB* mutant strain was constructed using P1 transduction [16] of JW3622 from the Keio collection of *E*. *coli* single gene deletions [17] into *E*. *coli* strain MG1655 using resistance to kanamycin for selection. The deletion was verified by PCR using primers 5’-ATGAAAGTATGGATGGCG-3’ and 5’-CTAAGGTTCAAAACCTGTGATC-3’.

Site-directed mutagenesis was performed on the *ligB*-encoding ASKA plasmid (pCA24N JW3622) to introduce a two base pair non-synonymous mutation into the *ligB* gene to swap AAA to GCA using the following primers: 5'-GCGATCTTTGGGTGCAGCCAGCAGTTGATGG-3’ and 5'-CCAAAGATCGCTACGTTCTCGCATCCACAG-3’. The mutation results in a single amino acid change of the conserved KxDG motif previously shown to eliminate the ligase activity of ligase B [2]. The mutation changes the lysine at position 124 for an alanine (K124A). The nucleotide changes were verified by DNA sequencing; no other changes were seen.

### Bacterial growth

All growth measurements, unless stated otherwise, were performed using a Bioscreen C spectrophotometer. Overnight cultures were diluted into M9 medium containing 0.4% glucose and grown, shaking, until reaching OD_600_ ~0.4. Cultures were diluted 100-fold and 150 μl aliquots were placed into wells containing 150 μl of M9 medium containing 0.4% glucose and the indicated concentrations of the agents to be tested. Optical density was recorded every 15 minutes at 450 nm or 600 nm, as noted, for 24 to 72 hours, as indicated. Lag times were determined by an extrapolation from a linear regression of maximal growth rate for each growth curve using a custom R program [18].

### Bacterial survival assays

For bacterial survival assays, except for those with cadmium, standing overnight cultures were diluted 100-fold into 10 ml of fresh Lennox Broth (LB) and incubated, with shaking, at 37°C to mid-logarithmic phase (OD_600_ of ~0.4). Cultures were then divided into five 1-ml aliquots to which hydrogen peroxide, mitomycin C, bleocin, or ciprofloxacin was added at the indicated concentrations. Samples were incubated, shaking, for an additional 30 minutes. Then, for the aliquots with hydrogen peroxide, 100 μl of 50 μg/ml catalase were added to quench the peroxide. Cultures were diluted serially in M9 salts (1.28% Na_2_HPO_4_, 0.3% KH_2_PO_4_, 0.05% NaCl, 0.1% NH_4_Cl) and a 100 μl aliquot of a 10^−5^ dilution (~ 3 x 10^3^ cells) was spread onto LB-agar and incubated overnight at 37°C. Colony forming units (CFUs) were enumerated after 16 hours. For the UV irradiation experiments, cultures were grown and diluted serially as above and spread onto LB-agar. Plates were dried at room temperature for 20 minutes, exposed to the indicated doses of UV light using a CL-1000 ultraviolet crosslinker, and incubated overnight at 37°C. CFUs were counted after 16 hours.

Because CdSO_4_ causes a precipitate in M9 medium, for experiments that measured bacterial growth with cadmium cells were grown as above except in a minimal growth medium consisting of 0.5 mM KH_2_PO_4_ and 50 mM Tris-(hydroxymethyl)aminomethane hydrochloride with 0.1% NH_4_Cl, 0.1% NaCl, 0.1% KCI, 0.02% MgSO_4_, and 0.01% (NH_4_)_2_SO_4_, final pH = 7.2 [19]. Glucose was filter sterilized separately and added to a final concentration of 0.4%. Growth was monitored spectrophotometrically using a Bioscreen C at 450 nm (as in Mitra *et al*. 1975 (17)).

### Antibiotic susceptibility assay

Minimum inhibitory concentrations (MICs) of antibiotics needed to block bacterial growth were measured three times using E-Test strips. According to the manufacturer’s instructions, the highest of the three measurements was the MIC.

### Fluctuation analysis

Strains were grown in LB medium at 37°C shaking overnight. 100 μl of culture were transferred to 10 ml M9 minimal salts and 100 μl of this dilution was transferred to 10 ml M9 minimal medium [20] containing 0.4% glucose. Cells were then grown overnight at 37°C with shaking. The overnight culture was diluted to approximately 5,000 cells/ml in 20 ml of M9 minimal medium containing 0.4% glucose. Sixty 200 μl aliquots were taken from this culture and transferred to 150 mm x 17 mm tubes, which were shaken at 37°C for 24 hours. 48 of these cultures were each separately spread on LB-agar containing rifampicin (100 μg/μl), and appropriate dilutions of the remaining 12 cultures were spread onto LB-agar to quantify CFU/ml. After 24 hours, colonies were counted and the mutation rate calculated using the Ma–Sandri–Sarkar Maximum Likelihood Estimation Method of the Luria-Delbruck fluctuation test with the FALCOR web tool [21,22].

### DNA replication assay

Overnight cultures in M9 minimal medium containing 0.4% glucose were diluted into fresh medium containing [methyl-^3^H]-thymidine (1 μL/mL of cells), with specific activity of 70–86 Ci/mmole (1 mCi/mL) and with 0.1 mM IPTG to induce *ligB* overexpression. Although the amount of radioactivity is such that it will be fully incorporated within a few hours (*i*.*e*., there is not a continuous infusion of fresh label), 100 μl aliquots were taken at 2, 4, 6, 8, 10, and 12 hours. Additional samples were taken at 26, 28, and 30 hours for the culture with *ligB* overexpression. [Methyl-^3^H]-thymidine incorporation was measured according to Sambrook *et al*. 1989 [23]. Briefly, as previously published [24], cell culture aliquots were passed through Whatman GF/C filters using a Millipore manifold vacuum apparatus. The filters were subsequently washed, dried, and submitted to scintillation counting. Simultaneously, samples were taken from a second set of cultures without [methyl-^3^H]-thymidine and the OD_600_ was measured to quantify bacterial growth. These samples were then diluted serially and spread onto LB-agar plates to quantify cell viability (except for the zero time point which was determined using the OD_600_).

### Live/dead microscopy

Cultures of *E*. *coli* MG1655 carrying plasmids pLigB or pEmpty were grown, shaking, in LB medium overnight at 37°C. The overnight cultures were diluted 100-fold into fresh LB medium and grown, shaking, at 37°C to OD_600_ ~0.1. IPTG (0.2 mM) was then added to induce *ligB* expression and the time 0 sample was taken. The cultures were returned to shaking at 37°C and allowed to grow for 2 hours with samples being taken 1 and 2 hours later. For each sample the OD_600_ was measured and cells were pelleted by centrifugation. The number of cells taken for each sample was adjusted using the OD_600_ measurement to be equal to 1 ml of ~0.1 OD_600_ culture. The supernatant was removed and cells were stained for live/dead microscopy using the LIVE/DEAD BacLight Bacterial Viability Kit per the manufacturer’s instructions. Cell count and cell length measurements were done on the microscope images using the analyze particles function in Fiji [25].

### Measurement of biofilm formation

*E*. *coli* MG1655 and the MG1655 Δ*ligB* mutant strains were grown as standing overnight cultures in LB at 37°C. The overnight cultures were diluted 100-fold into M9 minimal medium supplemented with 0.4% glucose. 100 μl of the diluted cultures were added to each well of a 96-well plate and incubated at 37°C. After 24 hours, wells were washed twice with distilled water. 125 μl of a 0.1% crystal violet solution were added to each well for 15 minutes. Wells were then washed with distilled water four times. The 96-well plate was allowed to dry at room temperature overnight. The next day, 125 μl of 30% acetic acid were added to each well and mixed with pipetting. The acid-solubilized biofilm solutions were transferred to a fresh 96-well plate. Absorbance at 550 nm was measured using a Tecan Infinite M200Pro plate reader.

## Results

### Ligase B is highly conserved throughout the Enterobacteriaceae family

**Fig 1:**
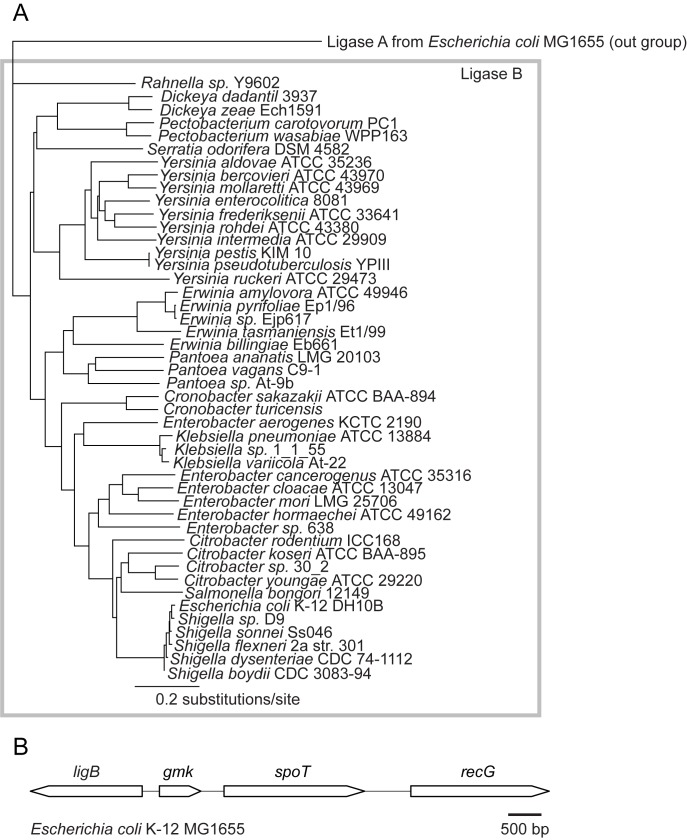
*E*. *coli* ligase B. (A) Ligase B amino acid sequence similarity tree. Ligase B peptide sequences from 46 species of Enterobacteriaceae are shown compared to the outgroup, ligase A. The distance of 0.2 substitutions per site is indicated. (B) The conserved *ligB* gene neighborhood (S2 Fig), represented by the region in *E*. *coli* MG1655.

We looked at 1,038 published *E*. *coli* genome sequences available from PATRIC [12] that ranged from laboratory K12 strains to multidrug-resistant clinical and environmental isolates. We found that *ligB* was annotated in all of them, with very low allelic variation, with an overall 97.9% nucleotide identity. The *ligB* homologs from 14 representative genera with complete genome sequences from within the *E*. *coli* family, Enterobacteriaceae, had 53.5% nucleotide identity, and maintained a 53.4% pairwise amino acid identity from the predicted translation of the coding sequence (S1 Fig). Strong conservation of sequence across multiple genera can be an indication of evolutionary pressure to retain function [26,27]. 46 species from the same 14 genera of Enterobacteriaceae were used to construct a phylogenetic tree of ligase B using ligase A from *Escherichia coli* MG1655 as the outgroup [28] (Fig 1A). We observed that ligase B maintains a tight phylogenetic grouping.

Conservation of gene order is uncommon in prokaryotes [29,30], yet we observed striking (14/14) synteny of the ~8 kb locus surrounding the *ligB* gene across multiple genera (S2 Fig). Additionally, in each of the 14 representative complete Enterobacteriaceae genomes, *ligB* and *recG* were mapped close together (ranging from 3,991 to 4,181 bp apart) and flanked the *spoT* gene, as depicted in Fig 1B. *ligB* also maintains a head-to-head juxtaposition with *gmk*, a gene that is upregulated in response to oxidative stress [31]. Conservation of gene order, orientation, and proximity can provide insight into gene function [30], suggesting that *ligB* may function to help the cell mitigate the effects of oxidative stress.

### *E*. *coli* lacking *ligB* is hypersensitive to hydrogen peroxide and mitomycin C

**Fig 2:**
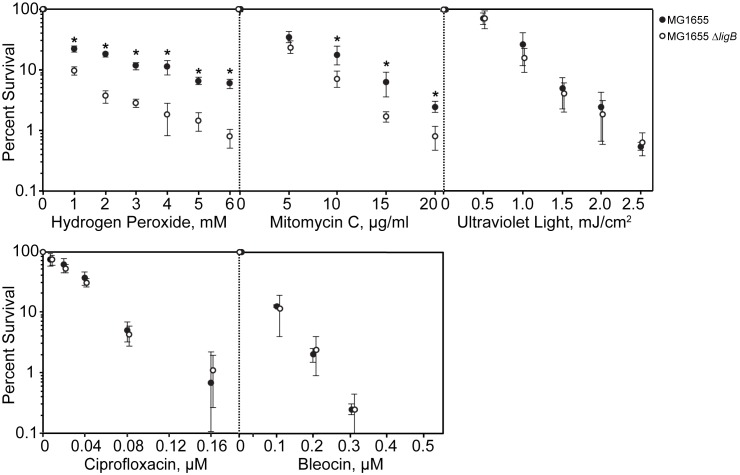
Effect of *ligB* deletion on bacterial response to DNA damaging agents. Survival of the parent strain MG1655 (●) compared to the isogenic Δ*ligB* strain (○). Percent survival was determined by dividing the number of CFU/ml with the indicated treatment by the number of CFU/ml without treatment and these data are shown on a semi-log plot as a function of increasing DNA damaging agent. Error bars denote the standard deviation calculated from experiments done in triplicate (repeated three times with similar results). Comparing the parent and the mutant strain for each specific condition by Student’s T-test, *p* < 0.05(_*_).

Treatment of *E*. *coli* with hydrogen peroxide, mitomycin C, UV irradiation, bleocin, or ciprofloxacin each induces specific types of DNA lesions that are repaired with various specific DNA repair mechanisms [32–36]. We deleted *ligB* in *E*. *coli* MG1655 and compared the survival of the mutant to the isogenic MG1655 parent strain when exposed to DNA damaging agents. There were up to nine-fold fewer Δ*ligB* colonies than isogenic parent colonies in the presence of hydrogen peroxide and four-fold fewer with mitomycin C (Fig 2). However, no survival differences were observed after exposure to UV irradiation, bleocin, or ciprofloxacin (Fig 2). A separate *ligB* deletion strain constructed previously by another group was found to have decreased survival upon exposure to nalidixic acid and methyl methanesulfonate, but the phenotype was subsequently found to be caused by Lula phage contamination rather than the *ligB* deletion [37]. Supernatants of saturated cultures of our *ligB* deletion strain were spotted onto uninfected cells with no evidence of Lula contamination [37]. Furthermore, compared to the isogenic parent, the "Δ*ligB*" (phi80) lysogen was dramatically more susceptible to UV irradiation [37] but the mutant we constructed and its isogenic wild-type parent strain were equally susceptible to UV irradiation. Therefore, phage contamination likely does not explain the phenotypes we observed for the Δ*ligB* mutant.

### Deleting *ligB* decreases MICs to cephalosporins and ciprofloxacin

Many antibiotics, including the fluoroquinolones, induce oxidative damage [38–40]. Although fluoroquinolone resistance is tightly linked to the G1264T *ligB* allele, the allele itself does not directly affect fluoroquinolone resistance [11]. To investigate the role of *ligB* in response to antibiotics, we examined the effect of *ligB* deletion on susceptibility to drugs representing six different antibiotic classes that either induce (top set, Table 1) or do not (bottom set, Table 1) induce oxidative damage. We observed MIC differences (≥ two-fold change indicated by gray shading in Table 1) for the two third-generation cephalosporins tested, cefotaxime (parent = 0.047 μg/ml; Δ*ligB* = 0.016 μg/ml) and ceftazidime (parent = 0.125 μg/ml; Δ*ligB* = 0.064 μg/ml), and the fluoroquinolone, ciprofloxacin (parent = 0.016 μg/ml; Δ*ligB* = 0.008 μg/ml). Deletion of *ligB* did not affect MICs of the other tested drugs. In common, in addition to causing increased reactive oxygen species, ciprofloxacin, cefotaxime, ceftazidime, and levofloxacin alter cell metabolism [39,41]. Levofloxacin MICs were unaffected by the deletion of *ligB;* this result may reflect the structural differences in these fluoroquinolones [42]. Nonetheless, the results suggest that ligase B may mitigate some of the cellular consequences of increased reactive oxygen.

**Table 1:** Effect of *ligB* deletion on antibiotic MICs.

	MICs (μg/mL)	
Antibiotic	MG1655	MG1655 Δ*ligB*
Amikacin	1.500	2.000
Ampicillin	1.500	1.500
Cefotaxime	0.047	0.016
Ceftazidime	0.125	0.064
Ciprofloxacin	0.016	0.008
Imipenem	0.250	0.250
Levofloxacin	0.020	0.020
Chloramphenicol	6.000	4.000
Ticarcillin/clavulate	2.000	1.500
Trimethoprim/ sulfamethoxazole	0.500	0.380

Top, antibiotics that generate reactive oxygen species; bottom, antibiotics that do not

### Absence of *ligB* increases mutation rate

Deletion of genes encoding proteins involved in DNA damage repair often result in an increased mutation rate of the organism [43,44]. In *E*. *coli*, acquisition of rifampicin resistance is a frequently used assay to measure mutation rate because alterations in 46 possible loci in the *rpoB* gene lead to a resistance phenotype [45]. The *ligB* deletion strain and its parent strain were exposed to rifampicin at 100 μg/μl and mutation rates were quantified using the Ma–Sandri–Sarkar Maximum Likelihood Estimation Method of the Luria-Delbruck fluctuation test with the FALCOR web tool [21,22]. The parent strain had a mutation rate of 1.8 x 10^−10^ per bp per cell (1.1 x 10^−10^–2.7 x 10^−10^, 95% confidence interval (CI)), which is similar to published mutation rates for *E*. *coli* MG1655 [46,47]. The *ligB* deletion strain had a mutation rate of 3.7 x 10^−10^ per bp per cell (2.6 x 10^−10^–4.9 x 10^−10^, 95% CI). This slight increase in mutation rate reveals that the Δ*ligB* strain is a weak mutator in the *E*. *coli* MG1655 background.

### Overexpression of *ligB* blocks cell growth

**Fig 3:**
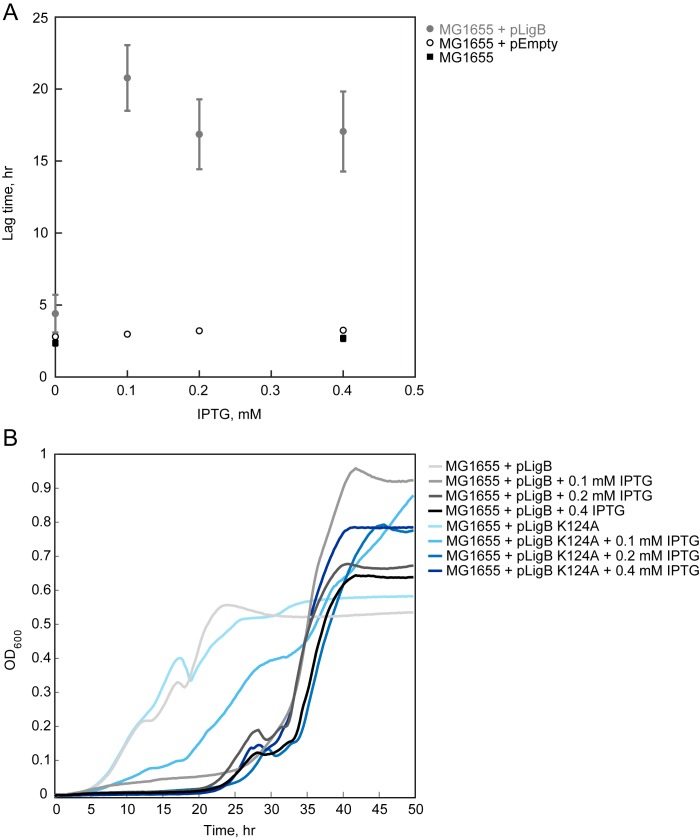
Effect of *lig*B overexpression on *E*. *coli* growth. (A) *E*. *coli* MG1655 either without (■) or with (●) plasmid pCA24N JW3622 [48] (pLigB), which encodes *ligB* under control of the T5 *lac* promoter, were grown in the absence or presence of IPTG. As a negative control, *E*. *coli* MG1655 with pCA24N [48] (pEmpty) (○), which is the parental “empty” vector, was included. Average lag time was quantified using a custom R program (21) and plotted as a function of IPTG concentration. Error bars denote the standard deviation calculated from experiments done in triplicate repeated at least two times. Where error bars are not shown, they were smaller than the symbol. (B) Representative growth curves of MG1655 with overexpression of pLigB and of ligase-deficient pLigB K124A with increasing levels of IPTG induction (lighter color (lower) to darker color (higher)). The experiment was repeated three times each in triplicate with the same results.

Growth of *E*. *coli* strain MG1655 transformed with a plasmid encoding *ligB* under control of the T5 *lac* promoter (pCA24N JW3622 [48]) (pLigB) was delayed in lag phase with the addition of IPTG. Lag phase time did not increase with increasing concentrations of IPTG (Fig 3A), indicating a threshold effect. After ~20–30 hours, the cultures regained normal exponential growth. Following recovery from the extended lag phase cells were re-induced with addition of fresh IPTG and the same growth block was seen. IPTG had no effect on cells harboring a plasmid encoding only the *lac* promoter (pEmpty). Furthermore, in the absence of IPTG, the strain harboring pLigB exhibited only a very slight increase in lag time compared to the control strains (Fig 3A). Thus, *ligB* overexpression blocked cell growth and caused a growth lag.

To determine whether the prolonged lag phase seen with *ligB* overexpression was dependent on the ligase activity of the enzyme, site-directed mutagenesis was performed on pLigB to mutate the lysine of the conserved KxDG motif to an alanine (K124A) thereby eliminating the ligase activity [2]. Although it took more IPTG, and, thus, possibly more *ligB* expression, to result in the maximal lag phase seen with wild-type *ligB* overexpression, overexpressed ligase-inactive *ligB* still caused the lag phase (Fig 3B). Therefore, the prolonged lag phase seen with overexpression of *ligB* was not strictly dependent on the ligase activity of ligase B, but less wild-type *ligB* appeared to be needed to cause the phenotype.

### Overexpression of *ligB* blocks DNA replication and kills cells

**Fig 4:**
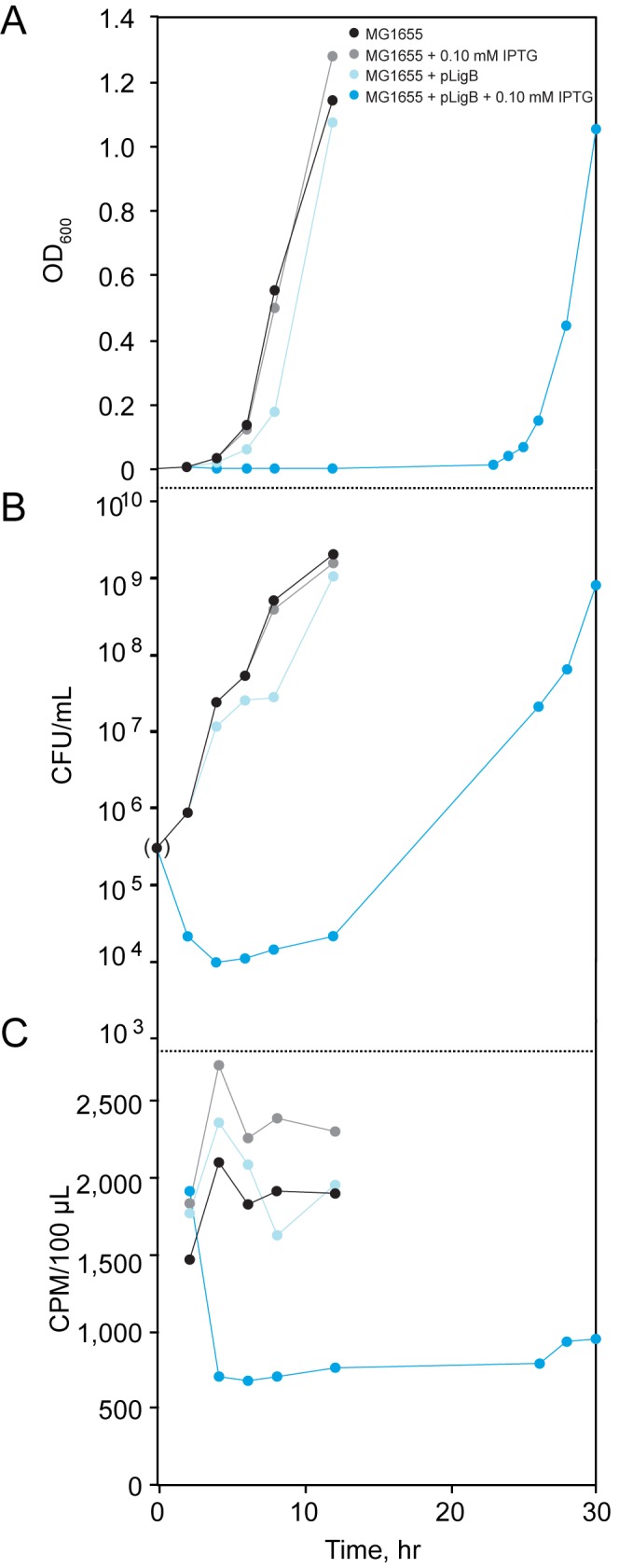
Effect of *lig*B overexpression on DNA replication and viability. The experiments shown in A,B, and C were performed concurrently. Effect of *ligB* overexpression on (A) bacterial growth, (B) cell viability (value in parentheses determined using OD_600_ value), and (C) incorporation of [methyl-^3^H]-thymidine. Lines connecting data points were added merely to aid visualization.

To test whether the extended lag phase seen with *ligB* overexpression affected DNA replication and/or cell viability, we quantified CFUs and [methyl-^3^H]-thymidine incorporation [49]. Simultaneously, bacterial cell growth (Fig 4A), cell viability (Fig 4B), and [methyl-^3^H]-thymidine incorporation were monitored (Fig 4C). The dramatic growth lag in cells containing the *ligB*-encoding plasmid induced with 0.1 mM IPTG is explained by a drop in cell viability (Fig 4B) and a block of DNA replication (Fig 4C). It is important to note that the label was not continuously added. Therefore, any recovery from the replication block would likely not be seen by this method; only a lack of incorporation for the cells overexpressing *ligB* can be concluded.

**Fig 5:**
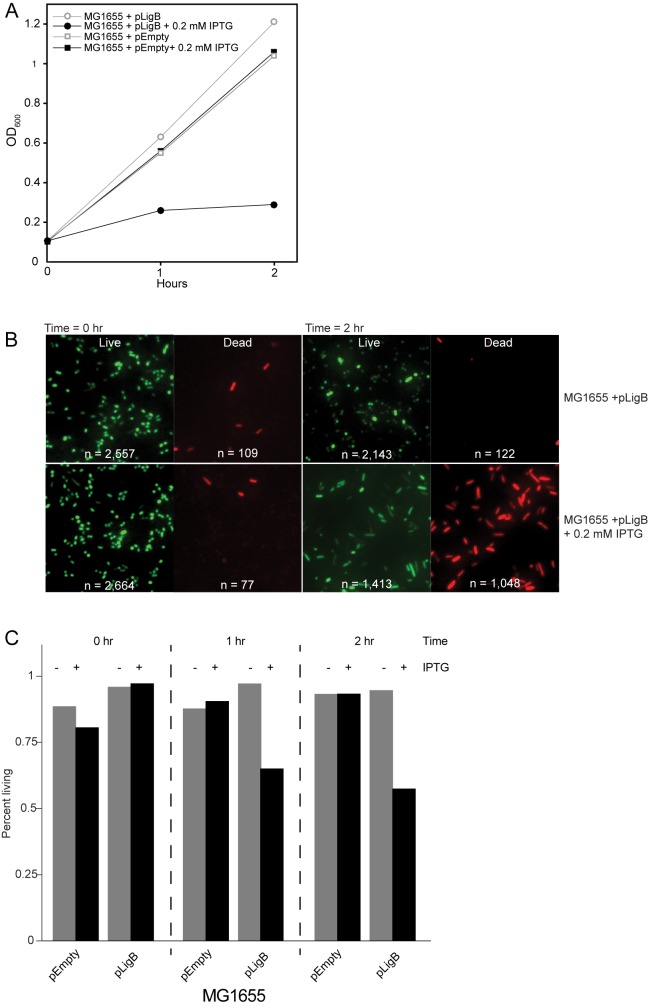
Effect of *lig*B overexpression during logarithmic phase. *E*. *coli* MG1655 with plasmid pLigB or pEmpty were grown overnight, diluted into fresh growth medium, grown to OD_600_ = ~0.1, and induced with 0.2 mM IPTG. At times 0, 1, and 2 hr after IPTG addition, the OD_600_ of the growing cultures was measured (A) and aliquots of each culture were submitted to fluorescent live/dead staining (B). Representative images are shown from two separate experiments with the same results. n is the total number of cells counted for each experimental condition. (C) Plot of the percentage of living cells for each strain at each time point post IPTG induction.

To test whether the replication block and reduction of cell viability also occurred with overexpression of *ligB* during the logarithmic phase of growth, cells were grown to early logarithmic phase (~0.1 OD_600_) and *ligB* overexpression was induced with 0.2 mM IPTG. To directly visualize the cells following induction of *ligB* overexpression, we used fluorescence microscopy and performed live/dead staining as well as quantified and compared cell lengths. Samples were taken at 0, 1, and 2 hours post induction. For each sample the OD_600_ was measured (Fig 5A) followed by staining and fluorescence microscopy (Fig 5B). With time, cell viability dropped; at the 2-hour time point, the viability of *ligB* overexpressing cells reduced from 97% to 57% (Fig 5C). The *ligB* overexpressing cells were elongated at two hours post IPTG induction compared to the cells harboring the empty vector (S3 Fig), supportive of a block in DNA replication in the surviving *ligB* overexpressing cells.

### Absence of *ligB* delays recovery following exposure to cadmium

**Fig 6:**
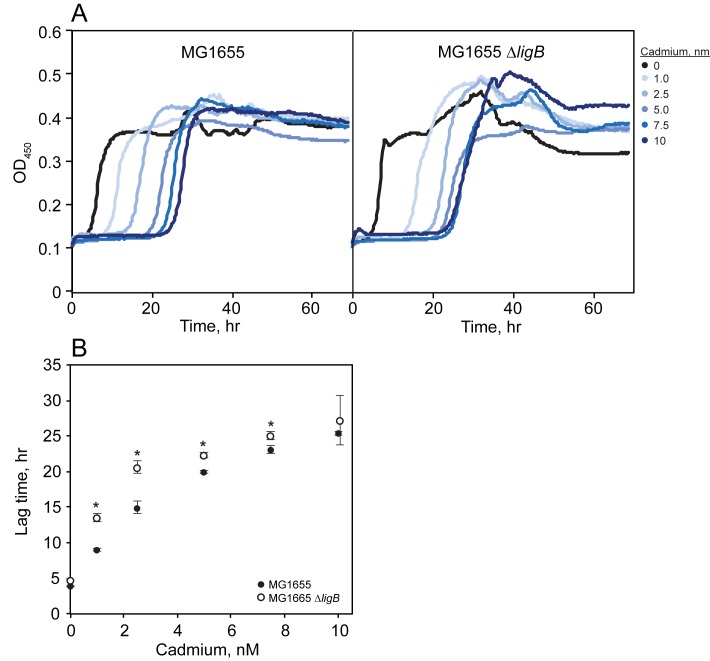
Effect of Δ*ligB* on growth in the presence of cadmium. (A) Growth of *E*. *coli* MG1655 or the isogenic Δ*ligB* strain was measured at OD_450_ [19] in the presence of the various indicated concentrations of cadmium. Growth without cadmium is in black and increasing cadmium is depicted from light blue (lowest) to dark blue (highest). The experiment shown was repeated three additional times (each in triplicate) with similar results. (B) Averaged growth lag times of *E*. *coli* MG1655 (●) or the isogenic Δ*ligB* mutant (○) as a function of cadmium concentration. Lag times were calculated with a custom R program [18]. Error bars denote standard deviation.

The growth delay phenotype of cells overexpressing *ligB* closely matches the growth phenotype of *E*. *coli* in response to the addition of cadmium, which causes single-strand DNA nicks [19,50]. This similar phenotype and the previous finding that the addition of cadmium to *E*. *coli* at pH 7 induces a three-fold increase of *ligB* transcripts [6] prompted us to compare the Δ*ligB* strain to its isogenic parent strain in response to cadmium. Compared to the isogenic parent, the Δ*ligB* strain was even further delayed in recovering from cadmium-induced damage at all tested concentrations of cadmium (Fig 6). The difference between the Δ*ligB* and the parent strain was greatest at the lower cadmium concentrations (Fig 6C). These data show that ligase B is not the direct cause of the increased lag phase phenotype of cells in response to cadmium. Instead, ligase B may function to help cells recover from cadmium damage.

### Absence of *ligB* reduces biofilm formation

Bacterial cells can develop resistance to antimicrobial agents by forming a biofilm. As mentioned above, *E*. *coli ligB* expression was previously found to be altered in biofilms [7,8]. To explore a potential role of *ligB* in biofilm formation, *E*. *coli* cultures were grown in 96-well plates for 24 hours without agitation. Washing and staining with crystal violet permits quantification of the biofilm retained in the plate by measuring the absorbance at 550 nm [51]. The Δ*ligB* strain had a 1.6-fold lower average OD_550_ (0.30 ± 0.05 compared to 0.48 ± 0.15 for the isogenic parent (*p* = 0.014)). By modulating biofilm formation, ligase B may contribute to antimicrobial susceptibility.

## Discussion

Hydrogen peroxide, mitomycin C, heavy metals, or UV radiation are toxic to *E*. *coli* [19,33,34,36]. These agents cause DNA adducts and other lesions that must be repaired for survival. Here we found that without *ligB E*. *coli* is more sensitive to hydrogen peroxide or mitomycin C, and more slowly recovers from cadmium exposure. At the same time, however, the absence of *ligB* had no effect on bacterial survival of ciprofloxacin, bleocin, or UV irradiation. Hydrogen peroxide produces oxidized DNA bases [34] and mitomycin C treatment results in the alkylation of DNA bases. Both of these types of damage are repaired by base excision repair or mismatch repair [52,53]. Cadmium inhibits the mismatch repair pathway [54], which may explain why the difference in lag time for the Δ*ligB* strain was most pronounced at lower cadmium concentrations (Fig 5). DNA damage caused by ciprofloxacin, bleocin, or UV irradiation is not repaired primarily through base excision repair or mismatch repair but through nucleotide excision or double-strand break repair [55,56]. This differentiation suggests that ligase B may function in base excision repair and/or mismatch repair.

A *ligB* allele associated with fluoroquinolone resistance modulates the survival of *E*. *coli* after exposure to hydrogen peroxide or UV irradiation [11]. Compared to its isogenic parent strain, we found that the Δ*ligB* mutant strain had lower cefotaxime, ceftazidime, and ciprofloxacin MICs. These antibiotics all increase the amount of reactive oxygen species within the cell to produce a pool of 8-oxoguanine [57]. Incorporation of 8-oxoguanine into the genome results in mismatched pairing of 8-oxoG-A. These 8-oxoG-A mismatches can result in mutation or death if not repaired [38,39,57]. Ligase B may, thus, function in mitigating DNA damage caused by 8-oxoguanine. Several other bactericidal antibiotics tested, however, such as levofloxacin, imipenem, and ampicillin can also produce increased reactive oxygen species, but the MIC for these antibiotics was not influenced by the deletion of *ligB*. Therefore, other factors possibly involving the GO system (*mutM*, *mutY*, and *mutT*) may be involved in how ligase B influences cell tolerance to different antibiotics.

The deletion of *ligB* had no effect on ciprofloxacin survival (as measured by CFUs (Fig 2)) yet affected the ciprofloxacin MIC. Thus, *ligB* deletion does not directly affect the bactericidal effects of ciprofloxacin but seems to only affect the bacteriostatic effects. The relative roles of generation of reactive oxygen species and stabilized topoisomerase-cleaved DNA to ciprofloxacin mechanism of action are unclear. Perhaps our finding of a distinction between the two with regards to *ligB* is a clue.

Antibiotic resistance development can be promoted by an increase in the mutation rate [58]. Also, bacteria in biofilms have increased antibiotic MICs as well as an increased likelihood for the development of additional antibiotic resistance [59]. We found that *E*. *coli* lacking *ligB* have a slightly increased mutation rate as well as a decreased ability to form biofilms. These changes may help explain the association of a *ligB* variant with fluoroquinolone resistance in *E*. *coli* clinical isolates when the variant itself does not directly increase fluoroquinolone MICs [11].

DNA nicks can lead to replication fork collapse and potentially lethal double strand DNA breaks [60]. Both the base excision repair and the mismatch repair pathways culminate in DNA nicks. Taken together, our data support the idea that ligase B either protects or directly ligates nicks resulting from exposure to DNA damaging agents that create lesions that are repaired through the base excision repair or mismatch repair pathways. Our finding that even a ligase-deficient *ligB* mutant was still able to cause increased lag phase highlights the importance of ligase B binding even in the absence of its enzymatic activity. DNA nicks may be sealed by ligase B directly or alternatively, ligase B binding may protect the nicks until ligase A ligates them. It is tempting to speculate, then, that without ligase B, DNA replication forks are more likely to run into unrepaired lesions, resulting in increased replication fork collapse, increased mutation, or death.

## Supporting information

S1 Fig Ligase B amino acid alignment of Enterobacteriaceae species. Clustal W alignment of ligase B from14 Enterobacteriaceae species with complete genome sequences aligned with the corresponding protein domains. The darkest shading is 100% and the lighter shading is 70% amino acid identity. The pairwise amino acid identity of this alignment, built using Jalview version 2 [S1 File], is 47.1%.
(PDF)

S2 Fig Synteny of *ligB* and neighboring genes in Enterobacteriaceae. The direction of and distance between the *ligB*, *gmk*, *spoT*, *and recG* genes for 14 different Enterobacteriaceae species with complete genome sequences are drawn to scale. The vertical dotted lines are to aid in visualization of the distances between genes and are set at the *E*. *coli* MG1655 distances as a reference.
(EPS)

S3 Fig Distributions of cell lengths with *ligB* overexpression. Cell length distribution plots for MG1655 harboring pLigB or pEmpty with and without IPTG induction at times 0, 1, and 2 hr post induction. This analysis was performed on images from one of two experiments with the same results.
(EPS)

S1 File Reference for supporting information. (DOCX)
