# Supplementary material for: Escherichia coli DNA ligase B may mitigate damage from oxidative stress
Source: PLoS One. 2017 Jul 11;12(7):e0180800. doi: 10.1371/journal.pone.0180800 (PMC5507437; doi:10.1371/journal.pone.0180800)
Supplement: S1 Fig — Clustal W alignment of ligase B from14 Enterobacteriaceae species with complete genome sequences aligned with the corresponding protein domains. The darkest shading is 100% and the lighter shading is 70% amino acid identity. The pairwise amino acid identity of this alignment, built using Jalview version 2 [S1 File], is 47.1%. (PDF) [file pone.0180800.s001.pdf]

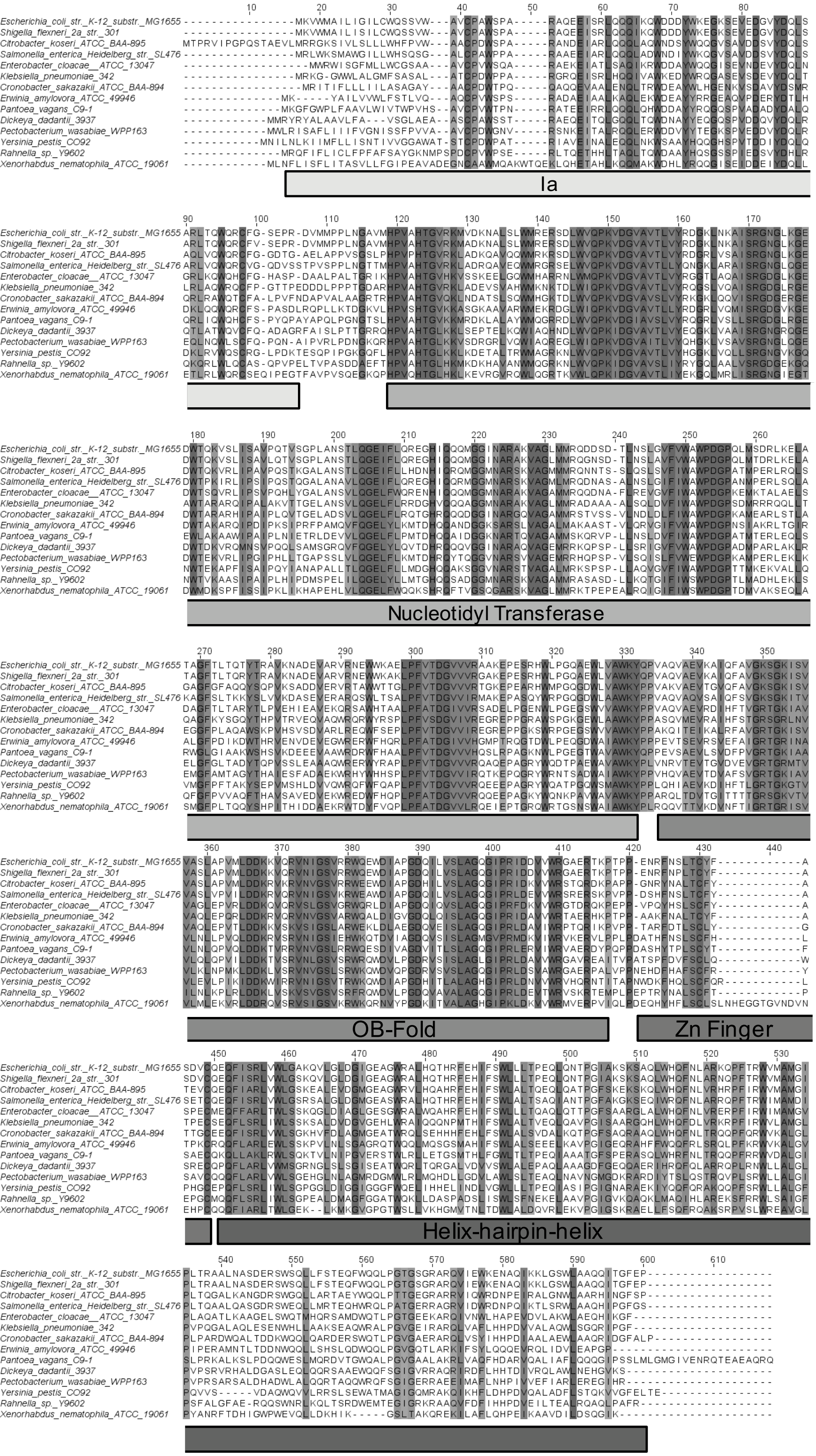

Escherichia_coli_str._K-12_substr._MG1655
Shigella_flexneri_2a_str._301
Citrobacter_koseri_ATCC_BAA-895
Salmonella_enterica_Heidelberg_str._SL476
Enterobacter_cloacae__ATCC_13047
Klebsiella_pneumoniae_342
Cronobacter_sakazakii_ATCC_BAA-894
Erwinia_amylovora_ATCC_49946
Pantoea_vagans_C9-1
Dickeya_dadantii_3937
Pectobacterium_wasabiae_WPP163
Yersinia_pestis_CO92
Rahnella_sp._Y9602
Xenorhabdus_nematophila_ATCC_19061
Ia
Nucleotidyl Transferase
OB-Fold
Zn Finger
Helix-hairpin-helix
