# Supplementary material for: Escherichia coli DNA ligase B may mitigate damage from oxidative stress
Source: PLoS One. 2017 Jul 11;12(7):e0180800. doi: 10.1371/journal.pone.0180800 (PMC5507437; doi:10.1371/journal.pone.0180800)
Supplement: S1 File — (DOCX) [file pone.0180800.s004.docx]

**Supporting information**

**Reference**

1. Waterhouse AM, Procter JB, Martin DMA, Clamp M, Barton GJ. Jalview Version 2—a multiple sequence alignment editor and analysis workbench. Bioinformatics. 2009;25: 1189–1191. doi:10.1093/bioinformatics/btp033
